# Integrated early childhood development policy in Iran: a stakeholder analysis

**DOI:** 10.1186/s12913-021-06968-2

**Published:** 2021-09-15

**Authors:** Omolbanin Atashbahar, Ali Akbari Sari, Amirhossein Takian, Alireza Olyaeemanesh, Efat Mohamadi, Sayyed Hamed Barakati

**Affiliations:** 1 Department of Public Health, Sirjan School of Medical Sciences, Sirjan, Iran; 2 Department of Health Management and Economics, School of Public Health, Tehran University of Medical Sciences, Tehran, Iran; 3 National Institute of Health Research, Tehran University of Medical Sciences, Tehran, Iran; 4 Health Equity Research Center (HERC), Tehran University of Medical Sciences, No. 70, Bozorgmehr Ava., Vesal St., Keshavars Blvd, 1416833481 Tehran, Iran; 5 Department of Global Health and Public Policy, School of Public Health, Tehran University of Medical Sciences, Tehran, Iran; 6 Population, Family and School Health Office, Ministry of Health and Medical Education, Tehran, Iran

**Keywords:** Early childhood, Development, Integrated policy, Stakeholder analysis

## Abstract

**Background:**

Many stakeholders are involved in the complicated process of policy making in integrated early childhood development (IECD). In other words, there are many challenges for IECD policy making in developing countries, including Iran. The aim of this study was to identify potential stakeholders and their interactions in IECD policy making in Iran.

**Method:**

A mixed-methods study was conducted in two phases in 2018. First, forty semi-structured interviews and a review of IECD-related documents were conducted to identify potential stakeholders and their roles. Second, using a designed checklist, these stakeholders were assessed for power, interest, and position in IECD policy making. Then, a map of stakeholders and a three-dimensional stakeholder analysis figure were designed.

**Results:**

The results of this study showed that various stakeholders, including governmental, semi-governmental, social, non-governmental and international organizations, potentially influence IECD policy in Iran. They were found to have diverse levels of power, interest and position in this regard, leading to their different impacts on the process. This diversity is assumed to have affected their levels of participation and support. Also, we found that the stakeholders with a high-power level do not have a high level of interest in, or support for, IECD policy. In general, organizational competition, complicated inter-sectoral nature of this process, insufficient budget, insufficient awareness about the importance of IECD, lack of priority given to IECD in relevant organizations, economical views rather than developmental perspectives, and lack of commitment among top managers are the reasons why this policy enjoys a low degree of support.

**Conclusions:**

There are weaknesses in effective interactions and relationships among IECD policy stakeholders. This will lead to the lack of equal opportunities for optimal early childhood development. To improve this process, advocacy from high-level authorities of the organizations, negotiation with child-friendly groups, establishing a body to coordinate and oversee children’s affairs, using the capacity of non-governmental organizations, strengthening inter-sectoral collaboration by clarifying the roles and responsibilities of stakeholders and the relationships between them, and increasing public awareness can be helpful.

## Background

Early child development (ECD) is a scientific approach to understand children’s growth and development in the early years and includes several areas which are likely to interact with each other. These areas are physical, cognitive, emotional, social, and spiritual domains [1]. Based on the definition given by the United Nations Committee on the Rights of the Child, 0–8 years of life is officially considered as the early years in ECD [2].

The idea of human development arose with the basic argument that human progress cannot be measured only by per capita income. In addition to having a good income, cultivating and developing human talents and capacities should be considered in this process. In fact, human development depends on many factors such as life expectancy, education, health, and environment [3]. Economically, Heckman compares the rate of return on investment for human development at different stages of life and shows that the higher rates of return on investment are observed in the early years in comparison with other stages of life. Later, the results of his study were confirmed by other economists [4].

Advancements in neuroscience also provided a scientific basis for re-emphasizing the vital importance of ECD. According to studies in neuroscience, brain develops at its fastest rate in early childhood, and this development, in addition to heredity, is influenced by many variables such as poverty [5], parental education [6], educational opportunities, quality of child care [7], social and cultural context [8], etc. Given the relationship between the developmental goals of nations and the quality of services for all child girls and boys and their families, ECD is becoming a priority for research, policy making, and planning [9]. However, equal opportunities are not available for all children to achieve their developmental potential, especially in developing countries including Iran. Moreover, there are inequalities among and within countries [10]. Based on the articles published in The Lancet, 200 million children under five years of age fail to reach their potential for cognitive development due to poverty, poor health, and inadequate nutrition and care [6].

The health system in Iran has a unique structure and governance in that the Ministry of Health and Medical Education (MoHME) is the stewardship of the health system [11]. The MoHME, through its extensive healthcare network which includes approximately 60 universities of medical sciences in 32 provinces of the country, aims to promote the health status of community by designing and implementing national health policies and programs. The universities of medical sciences are responsible for proving people living in their covered area with medical education, research and health services through the national healthcare network [12]. Although many efforts have been made over the past decades to improve children’s health in Iran, informants and executive authorities believe that in terms of normal development of children and related activities, still there are many challenges as far as the current status of Iran is concerned [13]. Actually, some development-related problems prevent children from enjoying their rights. Some of the most important challenges include the lack of a holistic and comprehensive view of child development, the low levels of social awareness [14], the small number of kindergartens and pre-schools so that a large number of children are still deprived of education, especially in remote and less developed areas, the quality and content of school curriculum in need of improvement [15], malnutrition among children in some parts of the country [14], and the lack of a national plan for this age range and defects in its implementation, with none of the previous development plans paying due attention to this area [16]. Therefore, governmental organizations did (and still do) not have any programs or instructions in this regard [13]. Although the MoHME offered an initial proposal for formulating a national document for IECD in 2008 by contribution from the Ministry of Education and the Social Welfare Organization [15], this document has not been implemented at a national level after several years. Therefore, there is no coherence and integration between the policies and programs of the main actors, with parallel work and sometimes conflicts between these policies. An important point in the continuity of this activity is to sensitize legislating and policy-making bodies to formally support IECD programs and their implementations.

Given the interdisciplinary and multidisciplinary nature of early childhood development, the participation of different stakeholders in any stage of the policy-making process is very important and their role in the success or failure of early childhood development policy is clear [2]. Even the best policies may fail at the implementation stage [17]. Therefore, effective policy-making requires identifying potential stakeholders and their characteristics and relationships [18]. Stakeholders are the individuals, organizations, and even governments involved in policy-making, the processes related to policy development and implementation, and the interactions between them [19]. In order to do a stakeholder analysis, tangible resources (financial resources, members, infrastructures, etc.) and intangible resources (expertise and legitimacy in the related issue, access to the media and political decision-makers) are examined and discussed, thereby evaluating their power. Their positions towards, and interests in, the issue and whether they are benefited or not from policy changes in this area are also identified. Finally, the stakeholder analysis map is drawn [20]. This study aims to identify potential stakeholders in ECD policies and determine the roles, positions, powers, interests and relationships that each of the stakeholders involved in ECD policies play or have in Iran to design appropriate policies and strategies. The purpose is to involve these key stakeholders in supporting IECD policies and make use of their potential capacities in IECD policy-making process.

## Methods

This study was conducted in two phases. In the first phase, a qualitative study was designed to identify potential stakeholders and explore their roles, powers, interests and positions in IECD policy making process. In the second phase, a quantitative approach was used to assess the powers, interests and positions of these stakeholders and draw a map as well as a three-dimensional analysis figure of the stakeholders.

### Phase 1: identifying potential stakeholders and their roles

#### Review of documents

Using a qualitative content analysis approach, the policy documents related to different fields of IECD including health, nutrition, education, early care as well as national upstream documents, laws, and international policies from 1964 to 2018 were reviewed in this study. The websites of several organizations such as the Ministry of Health and Medical Education, the Social Welfare Organization, the Ministry of Cooperatives, Labor, and Social Welfare, the Ministry of Education, the Parliament, Judicial System of Iran, the World Health Organization, the UNICEF and several other organizations were searched. In this line, the researchers resorted to personal and organizational relationships to identify and have access to relevant reports and documents. Finally, 64 national and 10 international documents and reports were identified and reviewed. Also, the literature was reviewed to identify all the relevant stakeholders and discover their roles, powers, positions, and interests.

#### Interviews

The other major method for data collection was face-to-face semi-structured interviews with purposefully identified policy makers (PM), managers (M), informed academics and researchers (Aca), NGOs’ representatives (NGO-R), and children’s service providers (CSP) in the child field from different organizations including the Ministry of Health and Medical Education, the Social Welfare Organization, the Ministry of Education, the Ministry of Cooperatives, Labor, and Social Welfare, the Ministry of Justice, Children’s Medical Center, the Parliament, the Society for Protecting the Rights of the Child (SPRC), the universities and the research centers, etc. Forty interviews each lasting for 30–90 min were conducted from October 2017 to June 2018 to reach data saturation. All interviews took place in the interviewees’ workplaces. The researchers used a literature-based and tailored interview guide. Before the interviews, necessary information regarding the study and its objectives were given to the participants and informed consent was obtained from them verbally. Moreover, they were assured that their information would remain confidential and the data of the study would be analyzed anonymously. The following questions were investigated during the interviews: What organizations, institutions, bodies, or persons are the potential stakeholders in IECD policy making? How do you see their roles, powers, interests, effects and interactions in this regard? Are they properly and adequately involved in the policy-making process? What are your suggestions for improving stakeholders’ collaborations in IECD policy-making process?

The interviews were transcribed verbatim and a thematic content analysis approach was used to analyze the data. Finally, to ensure the accuracy of our interpretations, the transcripts were shared with the participants who were asked to confirm their statements.

### Phase 2: assessing the powers, interests and positions of potential stakeholders in IECD policy-making process

In this phase of the study, we designed a checklist which contained the identified potential stakeholders and their roles in order to evaluate them in terms of power, interest and position. We asked the participants in the former phase to score each dimension. In this regard, the scores included numbers 1, 3, 5 and 7 (Table 1).

**Table 1:** Scores and dimensions in stakeholder analysis

score		1–3	5	7
**Dimension**	**power**	Low power	Medium power	high power
	**Interest**	Low Interest	Medium Interest	high Interest
	**position**	Low support	Medium support	high support

At this stage, 30 of the entire 40 checklists were returned. Then, the data were analyzed using Golden Grapher 13 and Microsoft Excel softwares. Finally, the researchers drew a map of stakeholders and a three-dimensional analysis figure of the stakeholders.

## Results

The results of this study are presented in different sections as follows:

### Potential IECD policy stakeholders

The identified stakeholders included the Ministry of Health and Medical Education, the Social Welfare Organization, the Ministry of Cooperatives, Labor, and Social Welfare, the Ministry of Education, the Judicial system of Iran, the Ministry of the Interior, non-governmental organizations, The Parliament, Children’s Service Providers, the mass media, the Planning and Budget Organization, universities, research centers and scientific association, families, international organizations (the UN, the World Health Organization, the UNICEF), the Intellectual Development Center for Children and Adolescents, high specialized councils (the Council for Higher Education, the High Council for Welfare and Social Security, the High Council for Health and Food Security), the Ministry of Agriculture, the Environmental Protection Organization, religious institutions, the Relief Committee, the Ministry of Industry, Mine and Trade, the Health Insurance Organization, the Ministry of Sport and Youth, the Ministry of Roads and Urban Development, The Police Force of the Islamic Republic of Iran, the Ministry of Communications and Information Technology, municipalities, the Ministry of Economic Affairs and Finance, the Ministry of Science, Research and Technology, and the Ministry of Culture and Islamic Guidance. In Table 2, these stakeholders, their potential roles and their mean scores in the dimensions of power, interest and position are shown.

**Table 2:** Determining stakeholders and their potential roles by documents reviews and interviews, and their mean scores in IECD policy in terms of power, interest and position by the designed checklist

Stakeholders	Potential roles	Power	Interest	Position
The Ministry of Health and Medical Education	Promoting children’s health through prevention, treatment and rehabilitation services	Medium-high	Medium-high	Medium-high
The Social Welfare Organization	Supporting homeless and poor families, and providing various services for children, including kindergarten services, support and care for children with special needs, orphaned children and children with disabilities	Medium	Medium	Medium-high
The Ministry of Cooperatives, Labor, and Social Welfare	Creating jobs, supporting the workforce including employed mothers, supporting and providing poor families with welfare services	High	Low- medium	Low- medium
The Ministry of Education	Providing education for children according to the pattern of cultural, religious, social, political and human beliefs and values	Medium-high	Low- medium	Low- medium
The Judicial System	Preparing judicial bills to protect children and the proper implementation of adopted laws related to children	Medium-high	Low- medium	Medium
The Ministry of the Interior	Policy-making and monitoring the performance of the provinces in order to implement the approvals in IECD policy	High	Low- medium	Low- medium
Non-governmental organizations	Participating in policy-making, and implementing voluntary affairs with cultural, charitable, environmental, humanitarian orientations for the development of children	Low- medium	High	High
The Parliament	Enacting laws and monitoring the implementation of laws related to children	High	Low- medium	Medium
Children’s Service Providers	Providing services to children in various fields including education, care and health	Low- medium	High	Medium-high
Mass Media	Informing and raising public awareness about early childhood development	Low- medium	Low- medium	Low- medium
the Planning and Budget Organization	Allocating budgets to the programs and continuous monitoring of the implementation of programs, their budgets, and their annual progress	High	Low- medium	Low- medium
Universities, research centers and scientific association	Training of human resources related to children, conducting research in various fields related to children, and publishing obtained information for policy-making, and raising awareness in the community	Medium	Low- medium	Low- medium
Families	Educating, nurturing, and providing necessary conditions for the optimal development of children	Low- medium	High	High
International organizations	Policy-making and helping to realize the rights of children, and improving their living conditions around the world	High	High	Medium-high
the Intellectual Development Center for Children and Adolescents	Involving in cultural works and products for children and adolescents	Low- medium	High	High
High specialized councils	Policy-making, designing macro strategies in specialized areas, and promoting inter-sectoral collaboration	High	Low- medium	Low- medium
The Ministry of Agriculture	Planning and policy-making to ensure food security of the community, and improving the quality of agricultural products	Low- medium	Low- medium	Low- medium
The Environmental Protection Organization	Protecting the environment, ensuring the correct and continuous use of the environment, and providing the conditions for sustainable development of the community	Medium	Low- medium	Low- medium
Religious institutions	Raising awareness, building the culture, and mobilizing material and spiritual resources of the community for ECD	High	Low- medium	Low- medium
The Ministry of Industry, Mine and Trade	Policy-making in the production of child-friendly products and licensing for businesses in the field of industry and commerce related to children	Low- medium	Low	Low
Health Insurance Organization	Providing affordable insurance coverage for families and children	Medium-high	Low	Low
The Ministry of Sport and Youth	Developing and promoting sports, physical activities and healthy recreation, and providing the necessary facilities and infrastructure in this regard	Low- medium	Low- medium	Medium
The Ministry of Roads and Urban Development	Adaptation of urban space, housing and roads for children in accordance with international standards with due attention to the safety of children	Medium-high	Low	Low
The Police Force of the Islamic Republic of Iran	Creating a safe environment for children’s development, and dealing with child abuse and other crimes	Low- medium	Low- medium	Low- medium
the Ministry of Communications and Information Technology	Policy-making in the domain of children’s use of information technology	Medium	Low	Low
Municipalities	Designing, planning and adapting urban environments and spaces for children	High	Low- medium	Medium-high
The Relief Committee	Providing relief and support for poor individuals and families	Low- medium	Low- medium	Medium-high
The Ministry of Economic Affairs and Finance	Participating in the economic and financial policies of the country, and allocating the necessary funds for current and development expenditures in cooperation with the Planning and Budget Organization.	Low- medium	Low- medium	Low- medium
The Ministry of Science, Research and Technology	Development of required science and research in various fields related to children’s affairs	Medium	Low- medium	Low
the Ministry of Culture and Islamic Guidance	Promoting culture and public awareness in areas related qizto ECD	Medium-high	Low- medium	Low- medium

### Interest analysis

Based on our results, the non-governmental organizations, the Intellectual Development Center for Children and Adolescents, the families, the Children’s Service Providers and the international organizations such as the UNICEF and the World Health Organization had the highest interest in IECD policy. However, their full capacity is not used in policy making. The Ministry of Health and Medical Education and the Social Welfare Organization are rated medium in the domain of interest. According to the interviewees, ECD is not a priority among high-level managers of these organizations, with only the middle level managers of these organizations being interested in this policy. On the other hand, stakeholders such as the Ministry of Cooperatives, Labor and Social Welfare, the Ministry of Education, municipalities, the Planning and Budget Organization, the Judiciary System, the Parliament, high specialized councils, the mass media, religious institutions, the Ministry of Economic Affairs and Finance, the Ministry of the Interior, the Ministry of Culture and Islamic Guidance, the Ministry of Science, Research and Technology, the Ministry of Agriculture, the Ministry of Industry, Mines and Trade, the Ministry of Roads and Urban Development, the Ministry of Communication and Information Technology, universities, scientific associations and related research centers, the Health Insurance Organization, the Relief Committee, The Police Force of the Islamic Republic of Iran, and the Environmental Protection Organization are among the organizations have low interest in IECD policy. The most important reasons for the lack of interest among some stakeholders, as stated in the interviews, include the lack of sufficient awareness of IECD and its importance thereby giving priority to other issues, considering children like other people in the community and not paying attention to their particular needs as unique members of the community, the lack of awareness of their own indirect role in ECD by affecting the environment in which children live, the large number of missions and tasks so that the development of childhood is overlooked among other missions, and the dominance of economic perspectives over developmental concerns.

*“The Ministry of Education shows low interest in ECD. For example, our kindergarten coverage rate is 10 %, and 90 % of children do not have access to these services at all because kindergarten and preschool education are not free and such educational services are not important in our country” (PM 21)*.

*“I want to say that there is insufficient understanding of the importance of ECD at the highest levels of the Ministry of Social Welfare or the Ministry of Health. For example, the implemented actions in the health children’s department do not achieve its goal because it is not a priority for the Ministry of Health” (PM 36)*.

### Power analysis

Participants rated the non-governmental organizations, the families, the Intellectual Development Center for Children and Adolescents, and the children’s service providers as the least powerful stakeholders in IECD policy. Among them, the Social Welfare Organization, universities, research centers and scientific associations, the Environmental Protection Organization, the Health Insurance Organization, the Police Force of the Islamic Republic of Iran, and the international organizations were classified as medium-level in terms of power. Interestingly, many organizations with low interest in IECD had high power. However, for various reasons such as the lack of structure, the lack of interest, the lack of commitment in senior managers, the conflicting interests, and the different views and approaches, they seemed not to have used their power in IECD policy-making process. Such organizations included the Ministry of Cooperatives, Labor and Social Welfare, the Ministry of Education, municipalities, the Planning and Budget Organization, the Judiciary System, the Parliament, high specialized councils, the mass media, religious institutions, the Ministry of Economic Affairs and Finance, the Ministry of the Interior, the Ministry of Culture and Islamic Guidance, the Ministry of Science, Research and Technology, the Ministry of Agriculture, the Ministry of Industry, Mines and Trade, the Ministry of Roads and Urban Development, the Ministry of Communications and Information Technology, and the Relief Committee.

*“Non-governmental organizations can play a very good role in this field. Unfortunately, in our country, these organizations are not very strong, and if they gain power, they will become semi-governmental organizations and will not be allowed to operate independently.” (M 7)*.

*“To implement the integrated early childhood development policy, the Ministry of the Interior can be considered as one of the most powerful stakeholders because this program will be implemented as pilot in the three provinces of Kerman, Tehran and Kermanshah. And well, the provincial governments under the Ministry of the Interior have a very effective role here.” (PM 12)*.

### Position analysis

Most of the identified potential stakeholders had medium or low support positions. Stakeholders like the high specialized councils, the Ministry of the Interior, the mass media, the Planning and Budget Organization, universities, research centers and scientific associations, the Ministry of Agriculture, the Environmental Protection Organization, religious institutions, the Ministry of Industry, Mine and Trade, the Health Insurance Organization, the Ministry of Roads and Urban Development, The Police Force of the Islamic Republic of Iran, the Ministry of Communications and Information Technology, municipalities, the Ministry of Economic Affairs and Finance, the Ministry of Science, Research and Technology, and the Ministry of Culture and Islamic Guidance were rated as having a low level of position. Additionally, the Ministry of Health and Medical Education, the Social Welfare Organization, the Ministry of Cooperatives, Labor, and Social Welfare, the Ministry of Education, the Judicial Power, the Parliament, children’s service providers, international organizations and the Ministry of Sport and Youth had a medium level of position. On the other hand, the non-governmental organizations, the families and the Intellectual Development Center for Children and Adolescents were among stakeholders with a high level of position. The participants stated that the main organizations involved in IECD policy, including the Ministry of Health and Medical Education, the Social Welfare Organization and the Ministry of Education, have not yet been able to show a strong supportive position toward this policy despite the fact that they work together to design a policy document for IECD. In this line, one of the important reasons mentioned in the interviews is that these organizations have spent most of their time and energy competing for program ownership or leadership. In some interviews, it is also mentioned that the position of most organizations is not appropriate as far as inter-sectoral issues are concerned.

*“There is a lot of competition between organizations involved in early childhood development policy and therefore there is no serious intention to support this policy and most of them show a low level position” (CSP 16)*.

*“This program has not so far been supported well in the country, and many stakeholders who can play a critical role in this regard, such as the Judiciary System, the Parliament, the Ministry of Education, the Ministry of Welfare, etc. have not paid much attention and support for early childhood development and related policies and programs.” (Aca 3)*.

**Fig. 1:**
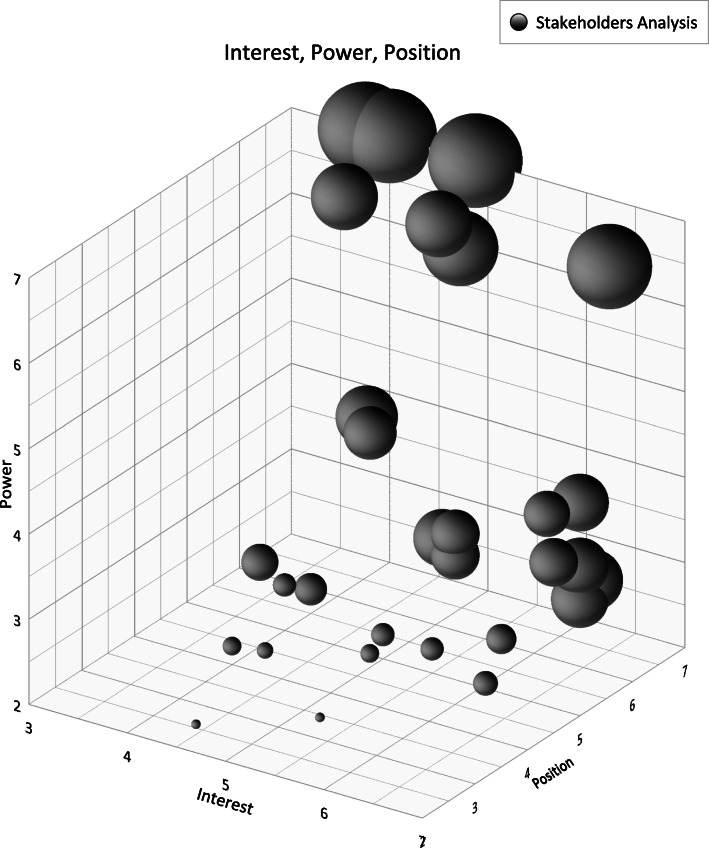
3-D Plot of Stakeholder Analysis

Also, in the three-dimensional stakeholder analysis figure (Fig. 1), the relationships among the dimensions of power, interest and position are shown. In this figure, the sizes of the plots indicate the powers of the relevant stakeholders. As can be seen in this figure, organizations with high power have a low-to-moderate interest and supportive position towards early childhood development policies and vice versa.

## Discussion

The results of this study show that many stakeholders play a role in ECD. The identified stakeholders have different interests, powers and positions towards IECD policy so that those with a high power do not have a high interest or position towards this policy and vice versa. Identifying stakeholders and their coalitions and interests in relation to ECD policies is crucial as this will clearly reflect the multiplicity of interests among various stakeholders, including those whose interests are threatened or reinforced by IECD policy. On the other hand, volunteers from different organizations have the time, capacity and resources to help this vulnerable community through stakeholders’ analysis which can be used to focus on their capacities [21].

Based on our study, although some stakeholders in the ECD policies such as the non-governmental organizations and the families have high interest in relation to ECD, their capacities and interests are not used in IECD policy-making process. The family, as the social institution in which the child grows up, has a very effective role in the development of children, and the best policies will fail if the families do not accompany the policies. In this regard, Sharpley emphasizes the role of parents as stakeholders in ECD policies. He states that parents have rights in terms of guidance, care and education of their children. They are also legal representatives who work for, and on behalf of, their children to the effect that they are required to provide the physical and emotional needs of their children including shelter, nutrition, health, education, and safety. Therefore, it is necessary to use their potential and interest in supporting early childhood development policies since the families’ interests and enthusiasm can be increased through these interactions and the parents’ peace of mind can be guaranteed. He also states that parents are one of the most important stakeholders who need workshops and training related to child development, which empowers them and helps them break negative cycles [22]. Based on the results of this study, family empowerment via educating the parents has been identified as a very important strategy for involving the family in IECD policy. On the other hand, the family as a social institution is located in the category of low-power stakeholders. Participants stated that families are only able to control their internal environment and are not able to control various factors affecting the development of children in the middle and macro levels. Families all around the world need government support to raise their children. This support can vary from financial and educational to security aspects.

According to the results of this study, various factors such as government ownership, lack of proper communication channels, and cumbersome administrative procedures and laws have prevented the use of the capacities of NGOs in the ECD policy process. Sharpley referred to NGOs as stakeholders that are familiar with ECD programs. He states that they can also participate in charitable activities such as providing toys and food which contribute to children’s development [22]. Also, Nene states that NGOs including community-based organizations, religion-based organizations, and bilateral and multilateral partners such as the UNICEF, the World Bank, the UNESCO, etc., provide support for early childhood education (ECE) policies. It can be said that NGOs and religious organizations complete the government’s efforts in mobilizing resources, building capacities for the ECE programs, and advocating through community sensitization [23]. Based on our findings, it is necessary to provide the context as well as the conditions so that the capacity of non-governmental organizations and the private sectors can be used in the policy-making process. In this regard, Woodhead and the National ECD Secretariat of Uganda emphasize the need for increasing public-private partnerships as an effective strategy to increase the involvement of other stakeholders and the community [2, 24]. In addition, Hamdy and Woodhead argue that not only should the potential of the NGOs, the community, and the municipalities be used in leading the early childhood development programs, but that the role of donors and NGOs should also be considered [2, 25].

According to the findings of our study, children’s service providers are ignored by the policymakers despite their high interest for participating in IECD policy-making process, and the top-down approach to policy-making in our country prevents them from influencing the policy-making process. However, according to available evidence, top-down and bottom-up approaches should be combined and used simultaneously so that we can employ the strengths of both approaches and cover their weaknesses. This approach allows us to use the capacity of street-level stakeholders in policy-making [26]. Also, in our study, international organizations are placed in the category of organizations with high interest and moderate power and position. The political commitment of international organizations to ECD can play an important role in facilitating national political commitments to young children. Powerful sponsors of investment in ECD include the UNESCO, the UNICEF, the World Bank, and the World Health Organization. These large organizations provide the leaders of the countries with financial support and technical advice such as the latest evidence and the best practices. In addition, international development conventions can support national and social policies that focus on the needs of children [27].

In terms of power and position, the results of this study indicate that most stakeholders with low-medium interest and position in IECD policy have high power in the policy-making process. This power originates from numerous factors including the number of resources (financial and human resources, and specialized knowledge), the ability to make use of these resources, the ability to influence other stakeholders, and legal legitimacy. However, as mentioned earlier, they have not used their power in IECD policy making for various reasons such as the lack of structure, the lack of interest, the lack of commitment in senior managers, the conflict of interest, and the different views and approaches. Strategies related to negotiation, advocacy, communication, promotion of inter-sectoral collaboration, and integrated approaches have been proposed to encourage these stakeholders to support the early childhood development policy. These strategies are mentioned in various forms in the literature. Since ECD has necessarily emerged as an inter-sectoral issue, and that an integrated approach essentially puts inter-sectoral collaboration systems on its agenda, countries seek an integrated approach to ECD policy planning that helps all sectors contribute to the improvement of children’s survival, growth, development, and success in school [22]. In this regard, Britto has proposed strategies such as the selection of a ministry for planning the policy making process, the creation of mechanisms for vertical and horizontal coordination and cooperation, and the adoption of coherent approaches [28]. Engle and Velea have also emphasized these strategies [29, 30]. The draft of the Republic of South Africa’s national early childhood development policy has noted the contributing role of policy strategies such as the compliance of sectoral policies, rules and programs among the various sectors responsible for ECD with the national ECD policy to ensure a national system for integrated and synergistic multi-sectoral ECD. Other strategies mentioned in this study include reforming the fragmented policy making and legislative framework of the ECD and delegating more responsibility to municipalities due to their capacity to cooperate with other government organizations to implement IECD policy [31]. In the meantime, Uganda’s National ECD Secretariat has also referred to the clarification of the roles of stakeholders to ensure accountability and minimize overlaps and parallel actions as well as the advocacy and negotiation for the inclusion of planning and budgeting of ECD interventions in sectoral programs and budgets [24].

## Conclusions

The results of this study show that due to the inter-sectoral nature of early childhood development and its broad scope, many potential stakeholders are influenced by this policy. Our results also show that the stakeholders who are in the category of high-power stakeholders do not have much interest or high supportive position towards this issue, which leads to a gap in this regard. The potential capacities and resources of all stakeholders, including NGOs, families, service providers, or street-level stakeholders, cannot be used due to this gap. Also, the power and influence of many stakeholders will not be used, which will result in the lack of equal opportunities for optimal development of children in the early years of life. Ultimately, the vital role of ECD in increasing human capital and sustainable development of society will be ignored. Based on our findings, strategies such as negotiation, advocacy, awareness-raising, empowerment, creating effective communication channels, adopting coherent approaches, promoting inter-sectoral collaboration, establishing leadership and coordinating organizations, using the capacities of street-level stakeholders, and promoting international cooperation can be effective in managing the stakeholders.

### Strengths and limitations

This study was done as the first study on stakeholder analysis of IECD policy-making process in Iran. The results of our study can be used for the management of potential stakeholders in this regard. However, our study had two main limitations; the first was the extensive research environment due to the inter-sectoral nature of the ECD and the diversity of groups involved in policy-making in this regard. The second was the difficult access to relevant experts and policy makers due to their busy schedules or lack of interest in participating in research. For this reason, a number of participants did not complete and return the checklists in the second phase of the study.

## Data Availability

Much of the data in this study are raw data, which are accessible to the researchers in the interviews and are reported in the paper. Also, the datasets used and/or analyzed during the current study are available from the corresponding author on reasonable request.
